# Downregulation of miR-145 contributes to lung adenocarcinoma cell growth to form brain metastases

**DOI:** 10.3892/or.2013.2728

**Published:** 2013-09-10

**Authors:** CHUNYANG ZHAO, YAN XU, YONGQIANG ZHANG, WEIWEI TAN, JIANXIN XUE, ZONGZE YANG, YOU ZHANG, YOU LU, XUN HU

**Affiliations:** 1 Biorepository, State Key Laboratory of Biotherapy, West China Hospital, Sichuan University, Chengdu, Sichuan 610041, P.R. China; 2 Molecular Medicine Centre, West China Hospital, Sichuan University, Chengdu, Sichuan 610041, P.R. China; 3 Department of Thoracic Oncology, Cancer Center and State Key Laboratory of Biotherapy, West China Hospital, Sichuan University, Chengdu, Sichuan 610041, P.R. China

**Keywords:** microRNA, primary lung adenocarcinoma, miR-145, brain metastasis, cell proliferation

## Abstract

The development of metastases involves the dissociation of cells from the primary tumor, penetrating the basement membrane, invasion and exiting from the vasculature to seed, and finally colonizing in distant tissues. The formation of brain metastasis (BM) in lung adenocarcinoma remains poorly understood. We examined the differential microRNA (miRNA) expression profiles of 5 primary and 3 brain metastatic lung adenocarcinoma samples by Agilent miRNA Microarrays. Five upregulated miRNAs (miRs-9^*^, -1471, 718, 3656, 720) and 3 downregulated miRNAs (miRs-214, -145 and -23a) were detected. The 4 most significantly deregulated miRNAs (miR-145, miR-214, miR-9^*^ and miR-1471) were validated in the additional 43 samples (35 primary and 8 brain metastatic lung adenocarcinoma samples) using TaqMan quantitative PCR. By functional assay, we found that the expression of miR-145 can regulate the ability of proliferation of A549 and SPC-A1 cells *in vitro*, but is not related to lymph node metastasis, migration and invasion. These results suggest that miR-145 may have a cell type-specific function and play important roles in the process of BM from lung adenocarcinoma.

## Introduction

microRNAs (miRNAs) are an endogenous conserved class of non-coding 20–24 nucleotide small RNAs that regulate gene expression at post-transcriptional level by mainly binding to 3′-UTR of target mRNAs, leading to mRNA degradation or translation inhibition (1,2). Bioinformatic analyses have predicted that 30% of human genes may be regulated by miRNA (3,4). miRNAs have been shown to regulate a variety of biological processes, including developmental timing, cell proliferation, cell differentiation and cell death (5,6). Several reports have elucidated the role of certain miRNAs as a class of oncogenes or suppressors of tumors, depending on their targeted genes (6–9). Moreover, accumulating evidence shows that miRNAs can influence multiple steps of metastasis, such as tumor cell migration, invasion and colonization and play an important role in tumor metastasis (10).

Brain metastases (BMs) generally tend to occur late in the progression of multiple types of cancer and are associated with poor patient survival. The BMs are the most common malignancies and occur in 20–40% of patients, an incidence 10 times greater than primary brain tumors, with a rising incidence in all countries (11,12). The most common tumors to metastasize to the brain originate in the lung, breast and skin (melanomas) (13,14). Lung cancer has the highest incidence for metastasis to the brain. Epidemiological studies have shown that approximately 15–30% of lung cancer patients develop BMs. The types of lung cancer with higher incidence of BMs are small-cell lung carcinomas (SCLC) and adenocarcinomas, and in more advanced cancer stages (15).

Metastasis, the spread of cancer cells from the site of primary tumor growth to distant organs, is a leading cause of cancer morbidity and mortality. The metastatic process is complex, requiring invasion from the primary tumor, intravasation, survival, arrest and extravasation of the circulatory system and colonization of a distant site (16,17). When tumor cells arrive at a new metastasis site, the vast majority of tumor cells that have undergone extravasation are still not able to effectively colonize the new site. For several types of carcinomas, solitary tumor cells can be detected in the bone marrow years before the development of overt metastasis (18). Thus, a better understanding of molecular factors that contribute to the growth and colonization of tumor cells in secondary sites is key in treating metastatic disease.

Molecules such as parathyroid hormone-related protein and transforming growth factor-β (TGF-β) that are produced by the cancer cells or are present in the bone microenvironment may mediate this growth (19). Some molecular factors that influence the ability of colon and other cancer cells to grow in the liver have also been identified, for example, the expression of the epidermal growth-factor receptor coupled with expression of growth factors in the tissue, such as transforming growth factor-α (TGF-α) (20–22). Reactive glia are recruited by highly proliferative BMs of breast cancer and promote tumor cell colonization (23). Expression of miR-200, which promotes a mesenchymal to epithelial cell transition (MET) by inhibiting Zeb2 expression, unexpectedly enhances macroscopic metastases in mouse breast cancer cell lines, enhances mouse breast cancer cell colonization to form distant metastases (24). These studies identified that some molecular and miRNAs can regulate growth and colonization of cancer cells in specific secondary sites.

To date, relatively little is known about mechanisms that enable BM and colonization of tumor cells, and even less is known about what causes it, such as miRNAs. To address miRNAs that regulate BM in lung adenocarcinoma, we compared the miRNA expression profiles between clinical human primary lung adenocarcinoma samples and BM samples from lung adenocarcinoma using Agilent Human miRNA Microarrays (25). In the present study, we showed that specific miRNAs may be involved in BM from primary lung adenocarcinoma. Furthermore, we demonstrated that the upregulation of miR-145 can suppress proliferation of human lung adenocarcinoma cells, but does not affect cell invasion and migration *in vitro*. These results suggest that miR-145 may play important roles for effective colonization of distant brain tissue sites by lung adenocarcinoma.

## Materials and methods

### Tissue samples

The 51 formalin-fixed and paraffin-embedded (FFPE) samples from 2 groups of cancer patients were studied, including 40 primary lung adenocarcinoma samples (20 samples with lymph node metastasis, 20 samples without) and 11 BM samples from lung adenocarcinoma. All samples were collected from West China Hospital, Sichuan University. A pathologist evaluated histologic tumor type, tumor grade, and tumor percentage using hematoxylin-eosin (H&E)-stained samples. Histologic classifications of the samples are summarized in Table I. Ethics approval for the study was obtained from the Ethics Committee of the West China Hospital of Sichuan University.

### RNA extraction and purification

Total RNA was extracted and purified using RecoverAII™ Total Nucleic Acid Isolation (Cat No. AM1975; Ambion, Austin, TX, US) following the manufacturer’s instructions and checked for an RIN number to inspect RNA integration by an Agilent Bioanalyzer 2100 (Agilent Technologies, Santa Clara, CA, USA).

### Microarray analysis

miRNA molecular in total RNA was labeled by miRNA Complete Labeling and Hyb kit (Cat No. 5190-0456; Agilent Technologies) following the manufacturer’s instructions, labeling section. Each slide was hybridized with 100 ng Cy3-labeled RNA using miRNA Complete Labeling and Hyb Kit (Cat No. 5190-0456) in hybridization Oven (Cat No. G2545A; Agilent Technologies) at 55°C, 20 rpm for 20 h according to the manufacturer’s instructions, hybridization section. After hybridization, slides were washed in staining dishes (Cat No. 121; Thermo Shandon, Waltham, MA, USA) with Gene Expression Wash Buffer kit (Cat No. 5188-5327, Agilent Technologies). Slides were scanned by Agilent Microarray Scanner (Cat No. G2565BA) and Feature Extraction software 10.7 (both from Agilent Technologies) with default settings. Raw data were normalized by Quantile algorithm, GeneSpring Software 11.0 (Agilent Technologies).

### TaqMan real-time PCR

Real-time quantitative PCR (qPCR) was carried out to quantify the mature miR-9^*^, miR-1471, miR-214 and miR-145 by TaqMan MicroRNA Assays according to the manufacturer’s protocol (Applied Biosystems, USA). The qPCR reaction was performed with the following parameter values: 15 min at 50°C, 10 min at 95°C, followed by 40 cycles at 95°C for 15 sec and 60°C for 1 min. The TaqMan probes were designed by ABI. miR-145, GUCCAGUUUU CCCAGGAAUCCCU; miR-214, ACAGCAGGCACAGAC AGGCAGU; miR-471, ACACCTGGCTCCACAGUGUGAC; miR-9^*^, UAAAGCUAGAUAACCGAAAGU; U6B, CGCAAG GATGACACGCAAATTCGTGAAGCGTTCCATATTTTT. qPCR was performed on ABI 7900 HT Sequence Detection System, and data were analyzed with 7900 System SDS software. U6B snRNA was used as normalization control. Relative expression values from 3 independent experiments were calculated following the 2^−ΔΔCt^ method.

### Cell culture

Human lung adenocarcinoma cell lines A549 and SPC-A1, and the adenovirus-immortalized human embryonic kidney epithelial cells HEK-293 were from the American Type Culture Collection and were maintained in DMEM (Gibco-BRL) with 10% fetal bovine serum. Cells were grown in a humidified atmosphere of 5% CO_2_ at 37°C.

### Recombinant adenoviral vectors for overexpression of miR-145

The pri-miR-145 sequence −179 to +287 (+1 being the first base of the mature miR-145) was amplified from HEK-293 cell genomic DNA. PCR product was cloned into pMD18-T (Takara Bio, Inc.), verified by sequencing, and subcloned into shuttle plasmid pAdTrack-CMV (designated as pAdTrack-miR-145) (26). pAdTrack-miR-145 linearized with PmeI was used to transform BJ5183-AD-1 cells harboring the adenoviral pAdeasy-1 vector for homologous recombination. Colonies were screened by plasmid miniprep and PacI restriction analysis to obtain clones with recombinant miR-145 (designated as pAdeasy-miR-145). PacI linearized pAdeasy-miR-145 was used to transfect HEK-293 cells to obtain packaged recombinant miR-145 adenovirus (designated as AD-miR-145). AD-miR-145 was amplified by repeated infection and verified by PCR. The pAdTrack-CMV empty vector was used as control (designated as AD-control). The titers and the multiplicity of infection were determined according to the manufacturer’s protocols.

### Cell proliferation assay

Cell proliferation was evaluated by Cell Counting Kit-8 (CCK-8) (Dojindo) according to the manufacturer’s instructions. Briefly, A549 cells (5,000 cells) and SPC-A1 cells (3,000 cells) were plated in 96-well plates in 100 μl/well and incubated for 24 h. Then, cells were infected with AD-miR-145 and AD-control (multiplicity of infection, 80). After 48 h, CCK-8 was added under sterile conditions, A549 cells were incubated for 2 h and SPC-A1 cells for 3 h before reading absorbance at 450 nm in an enzyme-linked immunosorbent assay plate reader (BioTeck Instruments, Inc.). Each experiment was performed in 4 replicate wells and independently repeated three times. Absorbance values were normalized to media control.

### Cell invasion and migration assay

The invasion assay was performed by using 24-well Millicell hanging cell culture inserts consisting of 8-μm PET membrane (Millipore) coated with BD Matrigel Basement Membrane Matrix. Briefly, the infected A549 and SPC-A1 cells were trypsinized and resuspended in DMEM containing 1% FBS and 5×10^4^ cells were added to the upper chamber of each well. After 24 h at 37°C, cells on the upper membrane surface were removed by careful wiping with a cotton swab and the filters were fixed by treatment with methanol for 20 min and stained with 0.1% crystal violet solution for 20 min. Invasive cells adhering to the undersurface of the filter were then counted (5 high-power fields) using an inverted microscope. The migration assay is the same as the invasion assay except no Matrigel was used.

### Statistical analysis

The SPSS 18.0 program was used for general statistical analysis. Data are expressed as means ± standard deviation (SD). Statistical significance of the studies was analyzed by Student’s t-test. To understand the relationship between miRNAs, the significant correlations were determined using the Kendall rank correlation test. P-value <0.05 was considered to indicate a statistically significant difference.

## Results

### miRNA expression profiling between primary and BM in lung adenocarcinoma

**Figure 1:**
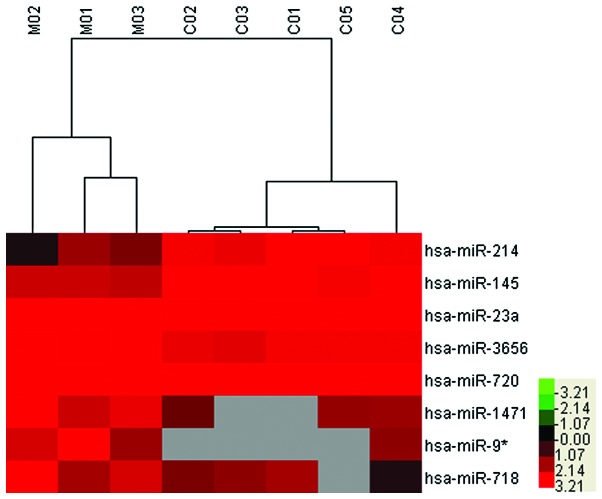
Two-way hierarchical clustering of 8 differentially expressed miRNAs. Primary lung adenocarcinoma: C01, C02, C03, C04 and C05; brain metastatic samples: M01, M02 and M03.

To investigate differentially expressed miRNAs between primary and brain metastatic lung adenocarcinoma, 5 primary lung adenocarcinoma samples without lymph node metastasis and 3 BMs in lung adenocarcinoma were analyzed using Agilent Human miRNA Microarray (8^*^60k) v16.0. The miRNAs that were altered by at least 2-fold and P-value was <0.05 were considered to be significant candidates. Heat maps depicted the relative expression level of mature miRNAs indicated by microarray analyses of the samples from 8 patients (Fig. 1).

Using strict criteria above, we identified 5 upregulated miRNAs and 3 downregulated miRNAs. miR-214 was the most downregulated miRNA with an average 20.33-fold change. miR-145 and miR-23a were also downregulated, with 6.9 and 3.62-fold change, respectively. We also observed an apparent increased expression of miRs-9^*^, -1471, -718, -3656 and -720. miR-9^*^ was the most upregulated miRNA with an average 64.84-fold change. All the differential miRNAs are shown in Table II.

### Verification of miRNA microarray data by TaqMan real-time PCR analysis

**Figure 2:**
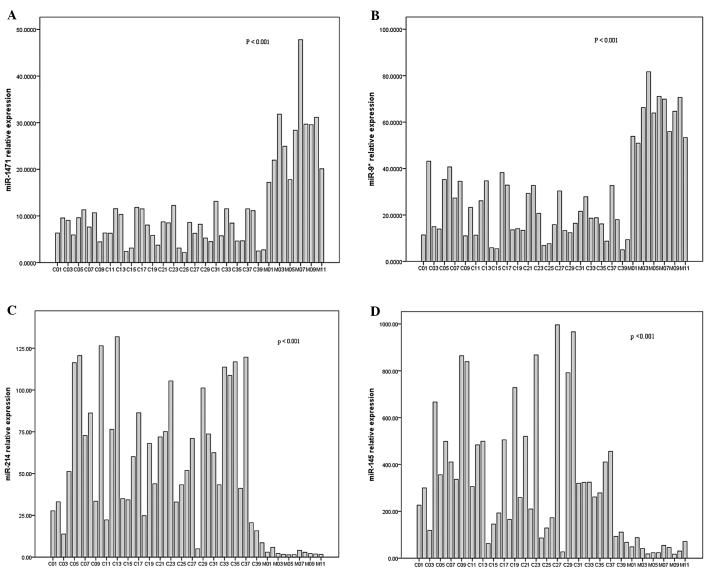
Validation of the 4 most significantly deregulated miRNAs, including (A) miR-1471, (B) miR-9^*^, (C) miR-214 and (D) miR-145. Every value represents the relative expression using the 2^−ΔΔCt^ method with U6B RNA as normalization control. Primary lung adenocarcinoma, C01–C40; brain metastatic samples, M01–M11. The order of the tissue samples is the same for all 4 plots.

To confirm our microarray data, TaqMan real-time PCR was performed to analyze the expression of the most significantly deregulated miRNAs, including miR-9^*^, miR-1471, miR-214 and miR-145, as shown in Table II. We examined the expression of 4 miRNAs in 51 samples (including 8 samples for microarray analysis). The miRNA expression level of each sample was quantified and normalized to U6B expression. The TaqMan real-time PCR data showed that the expression of miR-1471 and miR-9^*^ was upregulated significantly in BM samples vs. primary lung adenocarcinoma (p<0.001, p<0.001, respectively), with the increase of 3.65 and 3.14-fold, respectively (Fig. 2A and 2B). Consistent with the microarray data, as shown in Fig. 2C, miR-214 was downregulated significantly in BM samples with a decrease of 24.85-fold (P<0.001). miR-145 was also verified to be significantly downregulated 9.17-fold in BM samples (P<0.001, Fig. 2D).

### Bioinformatics analysis

We explored the role of the dysregulated miRNAs in primary lung adenocarcinoma progression and metastasis through bioinformatics analysis. Of note, a literature search showed that several of the miRNAs that we found dysregulated in BM from lung adenocarcinoma have also been reported to be altered in other types of tumor progression and metastasis (Table III).

To further evaluate the effect of these dysregulated miRNAs on the process of BM of lung adenocarcinoma, we focused on miR-145 and characterized its functional behavior first.

### Expression of miR-145 in primary lung adenocarcinoma with/without lymph node involvement

To investigate the relationship of the expression of miR-145 with lymph node involvement, we analyzed the relative expression level of miR-145 in 20 primary lung adenocarcinoma samples with and in 20 samples without lymph node involvement (including 5 primary lung adenocarcinoma samples used in microarray assay). However, the miR-145 expression level in our primary lung adenocarcinoma showed no significant difference between with and without lymph node metastasis.

### miR-145 overexpression inhibits the proliferation of human lung adenocarcinoma cell lines

**Figure 3:**
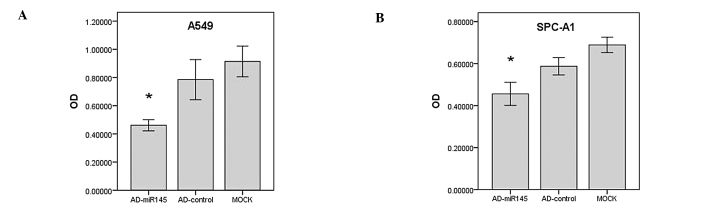
miR-145 overexpression inhibits the proliferation of (A) A549 and (B) SPC-A1 cells. CCK-8 assay reveal reduced cell growth for cell lines infected with AD-miR-145 compared to AD-control and MOCK (untreated cells) groups. The OD values are means of 3 separate experiments ± SD, and normalized to media control. ^*^P<0.05 by Student’s paired t-test compared to AD-control and MOCK groups.

One measure of the tumorigenic nature of cells is the ability to proliferate to form colonies in distant metastatic sites. To determine the effect of miR-145 expression on cell proliferation, we used AD-miR-145 and AD-control to infect human lung adenocarcinoma cell lines A549 and SPC-A1. After infection, we found the expression of miR-145 was increased in both cell lines that were infected with AD-miR-145 compared to AD-control and MOCK groups (data not shown). By CCK-8 proliferation assay, it was demonstrated that overexpression of miR-145 markedly inhibited cell proliferation in both cell lines. Compared to cells infected with AD-control, the average growth rate of A549 (Fig. 3A) and SPC-A1 (Fig. 3B) cells overexpressing miR-145 decreased by ~41 and 22%, respectively, in 3 separate experiments.

### miR-145 overexpression does not alter cell migration and invasion

**Figure 4:**
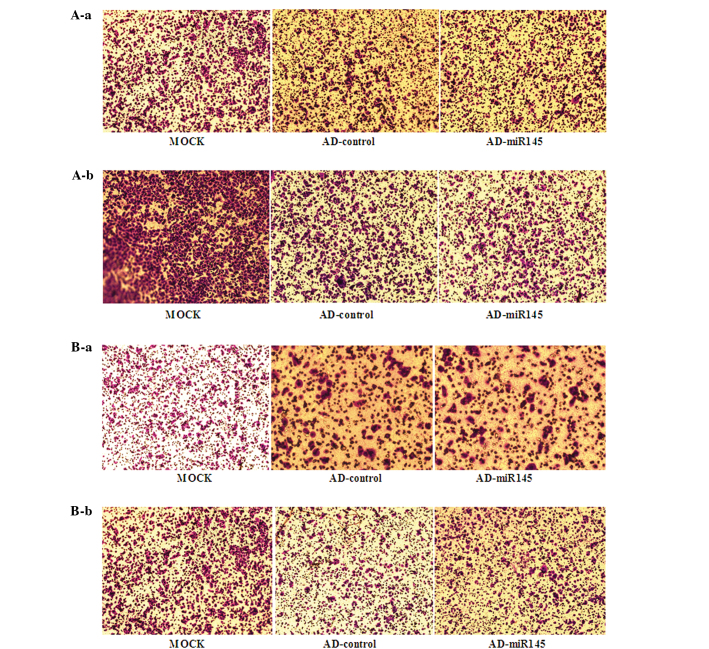
miR-145 overexpression does not affect cell migration and invasion ability of A549 and SPC-A1 cells. (A) Cell migration assay. (a) A549 and (b) SPC-A1 cells were transfected with AD-miR-145, AD-control and then subjected to transwell migration assays. After 18 h, migrated cells were counted after staining with crystal violet. (B) Cell invasion assay. (a) A549 and (b) SPC-A1 cells were transfected with AD-miR-145 and AD-control to perform transwell invasion assays. Following incubation for 24 h, invaded cells through the pores to the under surface of the membrane were fixed, stained and counted. MOCK, untreated cell group.

The TaqMan qPCR results showed that the expression of miR-145 in primary lung adenocarcinoma was not related to lymph node metastasis, suggesting that miR-145 may have no effect on the early stages of metastasis. To further determine the effect of miR-145 overexpression on the migration and invasion ability of A549 and SPC-A1 cell lines, the transwell assays were performed. Overexpression of miR-145 had no effect on migration (Fig. 4A) and invasion ability (Fig. 4B) of A549 and SPC-A1 cell lines.

## Discussion

Metastatic colonization of the brain has a poor prognosis and high mortality rates. To understand the molecular mechanisms that regulate the process of BM from primary lung adenocarcinoma, we initially compared the miRNA profiles between 5 primary lung adenocarcinoma samples and 3 BMs in lung adenocarcinoma samples by using Agilent Human miRNA Microarrays. In this study, 5 upregulated miRNAs (miRs-9^*^, -1471, -718, -3656 and -720) and 3 downregulated miRNAs (miRs-214, -145 and -23a) were significantly detected in brain metastatic tumors compared to primary lung adenocarcinoma. The 4 most significantly altered miRNAs from microarray were verified by using TaqMan real-time PCR with additional samples. Consistent with the microarray results, TaqMan real-time PCR results showed the expression of miRs-1471 and -9^*^ were significantly upregulated, but miR-214 and miR-145 were significantly downregulated in 11 BM samples compared to 40 primary lung adenocarcinoma samples. These miRNAs may be involved in BM from primary lung adenocarcinoma and have the potential to be biomarkers.

Downregulation of miR-145 was found in several tumor types including breast, gastric, lung, ovary, prostate cancer and esophageal squamous cell carcinoma (ESCC) (27–34). Accumulating evidence indicates that the processing of miR-145 is also involved in cancer metastasis. In breast cancer, miR-145 was identified to suppress breast cancer cell line invasion and metastasis by directly targeting MUC1 (35). miR-145-dependent regulation of 3′UTR of the JAM-A and fascin decreased motility and invasiveness of MDA-MB-231 and MCF-7 breast cancer cells (33). Using the prostate cancer cell lines, PC3 and DU145, miR-145 transfection can inhibit cell proliferation, migration and invasion by targeting FSCN1 (36). miR-145 is associated with bone metastasis of prostate cancer and may be involved in the regulation of EMT by reducing the ability of migration and invasion *in vitro*, and tumor development and bone invasion *in vivo* of PC-3 cells (37). In ESCC, by direct deregulation of FSCN1, miR-145 can inhibit cell proliferation and cell invasion in ESCC cells; it can also inhibit cell mobility (34,38). In addition, the high levels of expression of mature miR-145 were associated with recurrence of metastasis in ESCC patients (39). In bladder cancer, miR-145 also directly targets FSCN1 and inhibits bladder cancer cell line growth, migration and invasion significantly (40). These studies demonstrated miR-145 acts as a tumor suppressor in the progression and metastasis of cancer.

Our study demonstrated that miR-145 is downregulated in the BM compared to primary lung adenocarcinoma samples. Furthermore, we found that upregulation of miR-145 in lung adenocarcinoma cells suppresses proliferation of tumor cells. Consistent with our result, other reports also confirmed miR-145 can inhibit cell proliferation of human lung adenocarcinoma by targeting c-Myc, EGFR and NUDT1 (41,42). However, the transwell assays showed that upregulation of miR-145 has no effect on lung adenocarcinoma cancer cell migration and invasion. The miR-145 expression level also showed no significant difference between our primary lung adenocarcinoma samples with and without lymph node involvement. These findings suggest that the functional expression of miR-145 may also be cell type-specific. Considerable evidence indicates that molecular factors that are present in specific organs can, indeed, influence whether or not various types of cancer cells will grow there (43,44). The ability of cancer cells to grow in a specific site depends on features that are inherent to the cancer cell, features inherent to the organ and the active interplay between these factors. To colonize a new organ outright, disseminated tumor cells must have the capacity to productively interact with the new microenvironment in order to extract growth and survival advantages (45–47). Our studies suggest that downregulation of miR-145 should mainly contribute to growth and colonization advantages of lung adenocarcinoma cells at the metastatic site of the brain, but does not functionally support the involvement at early stages of metastatic disease (migration and invasion) in brain.

The formation of metastases requires the acquisition of genetic or epigenetic changes that allow for the detachment of cells from the primary tumor, transport and survival in the circulation and colonization in distant organs. We found that miR-145 is downregulated in the BM compared to primary lung adenocarcinoma samples. Our functional analyses showed that overexpression of miR-145 suppresses the growth of lung adenocarcinoma cells. These results outline an important role of miR-145 in the development of BM of lung adenocarcinoma and implicate its potential application in cancer therapy. To further understand the interaction between miR-145 and its targeted gene(s) in the BM of lung adenocarcinoma, functional characterizing of the target gene(s) is required in future studies.

## Figures and Tables

**Table I:** Patient characteristics.

Parameter	Primary lung adenocarcinoma samples (n=40)	Brain metastatic lung adenocarcinoma samples (n=11)
Age (years)	53.55±9.35	57.27±9.76
Gender		
Male	20	7
Female	20	4
LNM		
N0	20	
N1	4	
N2	14	
N3	2	
Differentiation		
II	15	
II–III	19	
III	6	

**Table II:** Differentially expressed miRNAs identified in brain metastasis from lung adenocarcinoma compared to primary lung adenocarcinoma by Agilent miRNA Microarray.

microRNA	Expression	Fold-change	P-value	Location
miR-145	Downregulation	6.9	0.0001	5q32
miR-214	Downregulation	20.33	0.021	1q24
miR-23a	Downregulation	3.62	0.022	19p13
miR-9^*^	Upregulation	64.84	0.008	1q22
miR-1471	Upregulation	26.05	0.017	2q37
miR-718	Upregulation	11.97	0.048	Xq28
miR-3656	Upregulation	4.38	0.046	11q23
miR-720	Upregulation	2.23	0.006	3q26

**Table III:** The involvement of miRNAs dysregulated in brain metastatic lung adenocarcinoma in the progression and metastasis of other types of cancer and their biological roles and targets.

miRNA	Deregulation	Cancer type	Biological function	Validated target genes
miR-214	Down	Ovarian cancer	Cell survival	PTEN
	Down	Cervical cancer	Cell growth and invasion	Plexin-B1
	Down	Breast cancer	Cell proliferation, invasion	Polycomb Ezh2 methyltransferase
	Down	Hepatocellular cancer		
miR-145	Down	Gastric cancer		
	Down	Hepatocellular carcinoma		
	Down	Lung cancer	Cell proliferation	c-Myc, EGFR, NUDT1
	Down	Ovarian carcinoma		
	Down	Colon cancer	Cell growth	IRS-1, YES, STAT1
	Down	Prostate cancer	Cell proliferation and migration, regulation of EMT	BNIP3
	Down	Breast cancer	Cell motility and invasiveness	MUC1, JAM-A, fascin
	Down	Esophageal squamous cell carcinoma	Cell motility and invasiveness	
miR-23a	Down	Hepatocellular carcinoma		
	Down	Lung cancer	Regulation of EMT	E-cadherin
	Up	Colon carcinoma	Cell growth, invasion and metastasis	MTSS1
	Up	Gliomas		CREB, PTEN
miR-9^*^	Up	Brain cancer		
miR-1471	Up	Rectal cancer		
	Up	Breast cancer		
miR-718	Up	Breast cancer		
