# [Expression of Concern] Downregulation of miR-145 contributes to lung adenocarcinoma cell growth to form brain metastases

**DOI:** 10.3892/or.2025.8991

**Published:** 2025-09-18

**Authors:** Chunyang Zhao, Yan Xu, Yongqiang Zhang, Weiwei Tan, Jianxin Xue, Zongze Yang, You Zhang, You Lu, Xun Hu

Oncol Rep 30: 2027–2034, 2013; DOI: 10.3892/or.2013.2728

Following the publication of the above paper, it was drawn to the Editor's attention by a concerned reader that, regarding the cell migration and invasion assay experiments shown in Fig. 4, the ‘Mock’ data panel in Fig. 4A-a (A549 cell line, migration assay) was apparently identical to the ‘Mock’ data panel in Fig. 4B-b (SPC-A1 cell line, invasion assay), even though the reported experimental conditions were different.

The authors were contacted by the Editorial Office to offer an explanation for this apparent data duplication; however, up to this time, no response from them has been forthcoming. Owing to the fact that the Editorial Office has been made aware of potential issues surrounding the scientific integrity of this paper, we are issuing an Expression of Concern to notify readers of this potential problem while the Editorial Office continues to investigate this matter further.

